# The Role of Obesity in Renal Cell Carcinoma Patients: Clinical-Pathological Implications

**DOI:** 10.3390/ijms20225683

**Published:** 2019-11-13

**Authors:** Gaetano Aurilio, Francesco Piva, Matteo Santoni, Alessia Cimadamore, Giulia Sorgentoni, Antonio Lopez-Beltran, Liang Cheng, Nicola Battelli, Franco Nolè, Rodolfo Montironi

**Affiliations:** 1 Medical Division of Urogenital and Head & Neck Cancer, European Institute of Oncology IRCCS, 20141 Milan, Italy; franco.nole@ieo.it; 2 Department of Specialistic Clinical and Odontostomatological Sciences, Polytechnic University of Marche, 60126 Ancona, Italy; f.piva@univpm.it; 3 Oncology Unit, Macerata Hospital, via Santa Lucia 2, 62010 Macerata, Italy; mattymo@alice.it (M.S.); giulia.sorgentoni@libero.it (G.S.); nicola.battelli@sanita.marche.it (N.B.); 4 Section of Pathological Anatomy, United Hospitals, School of Medicine, Polytechnic University of the Marche Region, 60126 Ancona, Italy; alessiacimadamore@gmail.com (A.C.); r.montironi@univpm.it (R.M.); 5 Department of Pathology and Surgery, Faculty of Medicine, 14080 Cordoba, Spain; em1lobea@gmail.com; 6 Department of Pathology and Laboratory Medicine, Indiana University School of Medicine, Indianapolis, IN 46202, USA; liang_cheng@yahoo.com

**Keywords:** obesity, body mass index, renal cell carcinoma, targeted therapy, immunotherapy, clinical outcomes

## Abstract

Obesity is a well-known risk factor for renal cell carcinoma (RCC) development. However, the RCC–obesity link has not been fully addressed when considering a comprehensive scenario starting from pathogenetic aspects through pathological issues up to the outcome of medical treatment. We therefore conducted an electronic PubMed search using keywords “obesity”, “body mass index”, “overweight”, “renal cell carcinoma/kidney cancer”, “medical treatment”, “targeted therapy”, and “immunotherapy/immune checkpoint inhibitors”. The selected data supported a crosstalk between adipose tissue (adipocytes and other white adipose tissue cells) and cancer cells inducing several signaling pathways that finally stimulated angiogenesis, survival, and cellular proliferation. Accurate sampling of renal sinus fat correlated with a prognostic value. Retrospective clinical evidence in metastatic RCC patients with higher body mass index (BMI) and treated with targeted therapies and/or immune checkpoint inhibitors showed advantageous survival outcomes. Therefore, obesity may influence the course of RCC patients, although the interplay between obesity/BMI and RCC warrants a large prospective confirmation. We are therefore still far from determining a clear role of obesity as a prognostic/predictive factor in metastatic RCC patients undergoing targeted therapy and immunotherapy.

## 1. Introduction

Renal cell carcinoma (RCC) is considered the most lethal cancer among the common genitourinary malignances [1]. RCC accounts for approximately 4% of all new cancers worldwide [2], and shows an increasing rate of incidence in the US, while the rate in Western Europe has been stable in the last decade. The majority of patients are males aged between 60 and 70 years.

Surgery, conservative where possible, is the current best practice for the treatment of localized or locally advanced RCC. It should be borne in mind that approximately 30% of patients who undergo partial or radical nephrectomy will develop distant metastases during their lifetime [2]. Furthermore, about 20% to 25% of RCC patients present metastatic disease at first diagnosis. Choosing the best therapeutic approach is fundamental in order to avoid unnecessary toxicities and optimize the outcome of RCC patients. In recent years, several agents have been developed that have substantially improved the prognosis of RCC patients. These agents target the vascular endothelial growth factor (VEGF; bevacizumab) and its receptor (VEGFR; sunitinib, sorafenib, pazopanib, axitinib, cabozantinib, lenvatinib, and tivozanib), mammalian target of rapamycin (mTOR; temsirolimus and everolimus), immunocheckpoints programmed death-1 (PD-1; nivolumab and pembrolizumab) and its ligand (PD-L1; atezolizumab), and cytotoxic T lymphocyte antigen 4 (CTLA-4; ipilimumab). Although major advances have been made in understanding the molecular basis of RCC carcinogenesis and metastatic spread, the choice of therapeutic sequence for each patient is still mainly based on clinical considerations.

Together with smoking habits and hereditary syndromes related to mutations in the *von Hippel-Lindau (VHL)* gene, obesity is a risk factor for RCC. Obesity is a worldwide debilitating disease, defined as a body mass index (BMI) exceeding 30 kg/m^2^, and characterized by a growth of white adipose tissue (WAT) [3]. Epidemiologically, it is well known that there is a close association of obesity with several non-cancer medical conditions such as glucose intolerance up to type 2 diabetes, dyslipidemia, metabolic syndrome, and cardiovascular diseases. In the field of cancer prevention, obesity is the second most common cause of carcinogenesis, after smoking [4]. Bearing in mind that the expansion of visceral WAT, which causes abdominal obesity, has been closely related to cancer cell growth [5], and assuming that by 2025 the worldwide obesity incidence is estimated to reach 21% in women, it is indisputable that the relationship between cancer and obesity is an extremely crucial health topic.

During recent decades, a large amount of data has been extensively analysed to investigate the impact of obesity/BMI on RCC occurrence, and as a result, a strong correlation in terms of carcinogenesis has been recognized, leading to obesity becoming one of the established and modifiable risk factors of RCC development both in men and women [6,7]. However, the relationship between obesity and RCC is still not completely understood for all stages of disease; in fact, studies on the relationship between obesity and RCC survival have yielded conflicting results [8,9]. Recent data underline that 40% of all cancer deaths in the United States are mainly caused by obesity [10], and an increased rate of obesity-induced mortality has been proved for many cancers, including RCC.

Recently, our group published a review article focusing on the role of obesity in genitourinary cancers with a particular focus on urothelial and prostate cancers. The available evidence underlined intriguing although often controversial results on the association of obesity/BMI with clinical outcomes of tumor response to therapies and survival outcomes [11].

Based on this scenario and taking into account the serious clinical-pathological implications arising, the current work examines the influence of obesity in metastatic RCC patients, focusing firstly on pathogenetic aspects concerning several signaling pathways, and then addressing pathological issues before examining the outcomes of targeted therapy and immunotherapy.

## 2. Article Selection

We conducted an electronic search of the PubMed database of the US National Library of Medicine, using keywords “obesity” or “body mass index” or “overweight” combined with “renal cell carcinoma/kidney cancer” along with “medical treatment” or “targeted therapy” or “immunotherapy/immune checkpoint inhibitors”. Gaetano Aurilio, Francesco Piva, and Matteo Santoni reviewed the most relevant articles published in English in conjunction with their references, and a selection was made for the present article. For article selection, priority was given to scientific articles published within the last 5 years.

## 3. Cancer–Obesity Link

White adipose tissue (WAT) is a complex cellular system harboring many other cells in addition to adipocytes, such as adipose stromal cells (ASCs), which fuel the endothelium and generate adipocyte progenitors [11], and a broad spectrum of innate and adaptive immune cells such as T and B lymphocytes, macrophages, dendritic cells, neutrophils, and mast cells. These cell types cooperate to produce proactive substances involved in the regulation of signaling pathways leading to carcinogenesis promotion.

**Figure 1:**
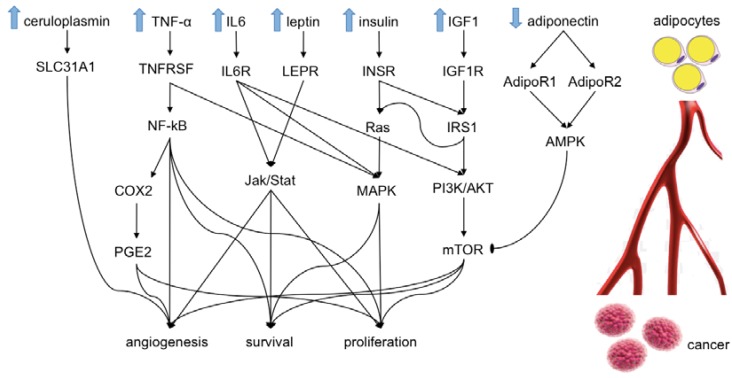
Pathways show the influence of adipocytes on cancer. The substances released from adipocytes diffuse through the blood circulation and reach cancer cells, where they activate different cellular pathways leading to an increase of cell proliferation, survival, and angiogenesis. IGF1: insulin-like growth factor I. IGF1R: insulin-like growth factor 1 receptor. INSR: insulin receptor. IRS1: insulin receptor substrate 1. BAD: Bcl2-associated agonist of cell death. MAPK: mitogen-activated protein kinase. LEPR: leptin receptor. ADIPOR1: adiponectin receptor protein 1. ADIPOR2: adiponectin receptor protein 2. VEGFA: vascular endothelial growth factor A. AMPK: AMP-activated protein kinase. mTOR: serine/threonine-protein kinase mTOR. CP: ceruloplasmin. SLC31A1: high-affinity copper uptake protein 1. TNF: tumor necrosis factor. TNFRSF: tumor necrosis factor receptor superfamily. IL6: interleukin-6. IL6R: interleukin-6 receptor subunit alpha. PTGS2: prostaglandin G/H synthase 2. PI3K: phosphoinositide 3-kinases. AKT: serine/threonine kinase. HIF1A: hypoxia-inducible factor 1-alpha.

Indeed, the biological cancer–obesity link is still yet to be fully defined, although a plethora of molecular mechanisms have been extensively investigated and postulated regarding the influence of obesity-driven biomarkers on cancer risk and progression. Possible biological mechanisms such as adipokines (leptin, adiponectin), insulin/insulin-like growth factor (IGF) pathways, chronic inflammation, and sex steroids have been implicated, along with various postulated molecules such as ceruloplasmin, tumor necrosis factor-α (TNF-α), interleukin-6 (IL-6), gastric inhibitory polypeptide (GIP), C-peptide, peptide YY, pancreatic polypeptide, and plasminogen activator inhibitor-1. These adipocyte-linked molecules come into direct contact with cancer cells via blood circulation, where they induce several cellular pathways, finally stimulating angiogenesis, survival, and proliferation of cancer cells [11]. Here we describe some of these pathways, as follows (Figure 1) [11].

A condition known as insulin-resistance does exist in obese patients and is associated with high levels of insulin-like growth factor I (IGF1) and blood insulin. Insulin-like growth factor 1 receptors (IGF1R) and insulin receptors (INSR) are generally up-regulated and, in turn, through the interaction with insulin receptor substrate 1 (IRS1) prime PI3K/AKT, mTOR/cyclin D1, mTOR/HIF1A/VEGF, and Ras pathways, finally induce cellular proliferation/angiogenesis and block apoptosis [12,13].

Moreover, obese patients have a high level of the hormone leptin, which interacts with its receptor (LEPR) stimulating cancer proliferation and survival by also involving MAPK, Jak/Stat, and PI3K/AKT pathways. Adiponectin in turn, through its receptors ADIPOR1 and ADIPOR2, has an anti-mTOR effect, thereby inhibiting the angiogenesis process [14]. Furthermore, obese patients exhibit high levels of ceruloplasim, an adipose tissue-induced substance, which interacts with its SLC31A1 receptor producing VEGF and consequently stimulating cancer angiogenesis [15]. Pro-inflammatory cytokines, in particular TNF-α and IL-6, trigger the production of cyclooxygenase 2 (COX2 or PTGS2), which in turn produces prostaglandin E2 (PGE2), favoring cancer progression. Of note, PGE2 may cause the release of inflammatory and angiogenic factors by cancer cells and hence contribute to changing the cancer microenvironment into an immunosuppressive environment. The activation of transcription factor NF-kb TNF-α increases anti-apoptotic factors such as BCL-2 and survivin. These, in turn, enhance cancer cell survival through an increase of cyclin D1 and cyclin E, which stimulate cellular proliferation, and the increase of several cytokines, e.g. IL-1, IL-2, and IL-6, with pro-inflammatory action. The complex IL-6/IL-6 receptor induces the PI3K/AKT pathway, which results in the enhancement of cancer cell proliferation and an anti-apoptotic effect.

Such crosstalk between adipose tissue (adipocytes and other WAT cells) and cancer can be summarized by the following sequence of steps: (i) in obese patients, cancer induction can be elicited via chronic inflammation in fat tissue and inflammation evoked by cytokines and chemokines as well as extracellular matrix enzymes, which in turn damages the genetic information within the cells causing mutations resulting in carcinogenesis; (ii) fat-tissue-derived cells that infiltrate cancer cells release adipokine signaling that can power cancer development; (iii) both adipocytes producing fatty acids/metabolites and the immune system dysregulation together can promote cancer aggressiveness [16]. Cellular interactions are regulated via adipocyte-induced paracrine and contact signals, while the stimulation of the innate and adaptive immune system comes about through adipocyte death generated by compromised oxygen supply [17]. To substantiate how a biological mechanism supports obesity-related cancer development, we propose the adipose fatty acid binding protein (A-FABP), which is another critical mediator predominantly expressed in mature adipocytes and involved in lipid transport, intracellular modulation of lipid metabolism, and gene expression regulation. Elevated human serum levels of A-FABP were observed in obesity [18], and correlated with breast cancer growth [19]. Of interest, Hao and co-workers used human samples and mouse models to show that (i) increased circulating A-FABP levels were found in obese patients with breast cancer; (ii) circulating A-FABP induced breast cancer aggressiveness in both in vitro and in mouse models; (iii) A-FABP promoted breast cancer stemness through the IL6/STAT3/ALDH1 signaling pathway; (iv) A-FABP ablation decreased obesity-associated breast cancer growth in different mouse models [20]. This study provided novel evidence for the linkage between obesity and breast cancer development, as further emphasized by Greenhill on Nature Reviews Endocrinology in October 2018 [21], and underlined the activity of common signaling pathways across different cancers.

## 4. Pathological Assessment and Prognostic Value of Adipose Tissue in RCC

According to the AJCC/TNM 8th Edition, invasion of perirenal and/or renal sinus adipose tissue by a kidney tumor should be considered as pT3a stage, independently of tumor histotype or tumor dimension [22,23,24]. The extension beyond Gerota’s fascia, including contiguous extension into the ipsilateral adrenal gland, should be considered as pT4 stage [22,23,24]. As a consequence of this staging system and of the prognostic value of perinephric fat invasion, accurate sampling of the perirenal and renal sinus fat is of great importance. Special attention should be paid to the assessment of perinephric fat invasion. Macroscopically, a pushing border with smooth convex outer surface, even if beyond the normal kidney, is not diagnostic of fat invasion. Loss of smooth interface or irregular nodules protruding into fat are likely to be diagnostic of invasion of perinephric fat. In such case, multiple perpendicular sections of the tumor fat interface are necessary. At microscopic examination, tumor cells touching adipose tissue or tumor nests extending as irregular tongues into fat, with or without desmoplasia, are diagnostic aspects of invasion [23,24,25].

**Figure 2:**
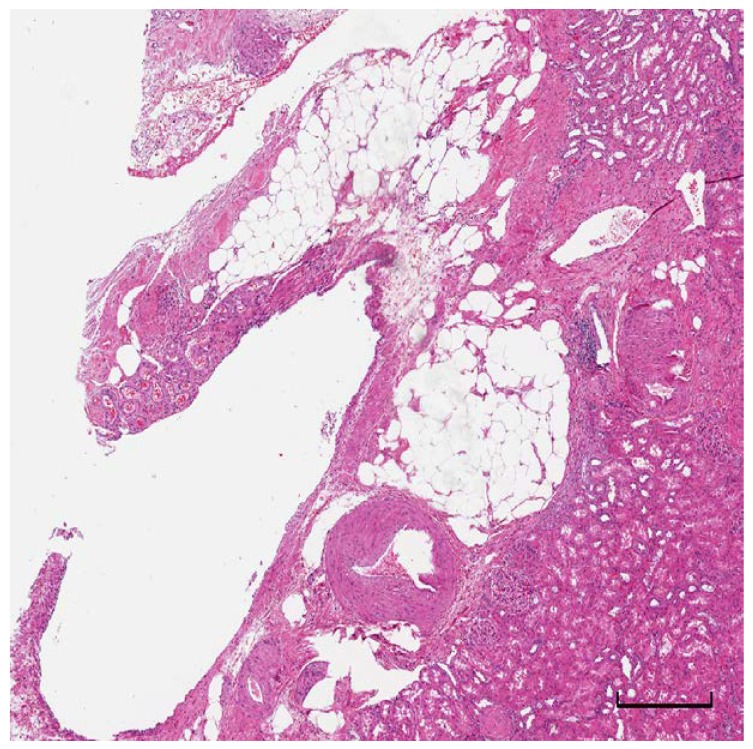
Proliferation of adipose tissue in renal sinus lipomatosis. Scale bar: 100 microns.

The renal sinus is the adipose tissue compartment located within the confines of the kidney not delimited from the renal cortex by a fibrous capsule surrounding numerous veins and lymphatic vessels. The proliferation of adipose tissue in this compartment configures a lesion called renal sinus lipomatosis, characterized by benign proliferation of fat and atrophy of the renal parenchyma (Figure 2). It is often associated with chronic pyelonephritis, renal lithiasis, and hydronephrosis, and occurs with advanced age, obesity, and exposure to steroids [26,27].

Since there are numerous lymphatic and renal vein tributaries in the sinus adipose tissue, invasion into this compartment may permit the dissemination of a tumor otherwise regarded as renal-limited. Indeed, the renal sinus has been recognized as the main route for extrarenal extension, and chances of sinus invasion increase with larger tumor size, particularly for tumors larger than 4 cm [28,29,30]. Renal sinus invasion is most commonly seen in clear cell RCC [31]. Of note is the fact that in clear cell RCCs ≥ 7 cm in their greatest dimension, renal sinus invasion is seen in more than 90% of cases. These observations have also been confirmed for papillary RCC and chromophobe RCC, although the involvement of the renal sinus was found to be less frequent in these two tumor histotypes. Even in carcinomas that appear to be renal-limited (pT1/pT2), sinus fat invasion can be present and is associated with risk of metastases [28,32,33].

In some cases, tumor bulging into the sinus can be difficult to interpret. Proper sampling of the renal sinus even for seemingly localized tumors is of great relevance. When invasion of the renal sinus is uncertain, at least three blocks of the interface should be taken. If sinus invasion is grossly evident, or obviously absent, one block is sufficient to confirm the macroscopic impression. Direct contact with renal sinus fat, or loose connective tissue, clearly beyond the renal parenchyma indicates renal sinus invasion, and any endothelial-lined space within the sinus, regardless of size, must be considered as renal sinus invasion [23].

Involvement of renal sinus fat appears to indicate a worse prognosis than involvement of only perirenal adipose tissue. Thompson et al. analyzed 205 pT3a clear cell RCC patients and found that patients with renal sinus fat invasion were 63% more likely to die of RCC compared with those with perinephric fat invasion, and that the risk of death persisted in multivariate analysis after adjusting for regional lymph nodes and distant metastases [29]. These results were confirmed by Bertini et al., who found a 5-year cancer specific survival (CSS) of 71.9% for pT3a/N0/M0 RCC patients with perinephric fat invasion only compared to 45.5% CSS for patients with sinus fat invasion [34]. As the relevance of this parameter has been described only recently, retrospective studies conducted on material without adequate sampling of the renal sinus should be considered with caution [35,36].

## 5. Obesity and RCC Risk

Epidemiological evidence collected during the 1990s highlighted a closer connection of obesity with RCC in females [37]. However, more recently, this relationship has been documented equally in both sexes [38]. A large prospective study was conducted over 8 years in patients in the United States assessing the relationship of RCC occurrence with weight measurements at predefined time-points. The results showed an association between baseline BMI and RCC risk, with further risk when documenting an increased BMI at age 50. Interestingly, weight gain equal to or exceeding 20 kg from younger age to 35 years or from age 35 to 50 was significantly correlated with RCC risk [39].

In 2013, Dobbins and co-workers published the results of a meta-analysis based on 98 observational RCC studies from 1985 and 2011 in which the association between obesity and cancer risk was assessed [40]. In comparison with people with normal BMI, the authors determined the relative risk of RCC as 1.57 in obese men and 1.72 in obese women, respectively [40]. Of interest, in a large Swedish male population, the relative risk of RCC occurrence was 1.8 and increased in persons presenting a BMI progressively higher by 15% over a range of 6 years compared with those without body weight variations [41].

The importance of BMI as risk factor has also been demonstrated by the attempt to incorporate it into novel stratification criteria for RCC patients treated with immune checkpoint inhibitors. Martini et al. [42] retrospectively reviewed a patient population mainly treated with anti-PD-1 as monotherapy, in which three variables, namely BMI, monocyte-to-lymphocyte ratio (MLR), and metastatic sites, were selected for identifying poor-risk patients (BMI < 24, metastases > 2 with liver metastases, and MLR > 0.93) and good-risk patients (BMI > 24, MLR < 0.93, and metastases < 2). The results demonstrated that in poor-risk patients, both overall survival (OS) and progression-free survival (PFS) were significantly shorter than in good-risk patients, suggesting that the variables used are promising factors for predicting survival [42].

Emerging data indicate a causal relationship between methylation of obesity-genes and RCC occurrence. Interesting data from a panel of 20 obesity-correlated genes from kidney biopsy samples demonstrated that patients with high levels of leptin receptor methylation were at risk of increased recurrence with consequent significantly shorter recurrence-free survival with respect to patients with low levels of leptin receptor methylation [43]. Most recently, Wang et al. [44] published a retrospective case-control study in which the association between circulating levels of a panel of 14 obesity-related biomarkers and clear-cell RCC risk was assessed. The findings highlighted that individuals with high levels of C-peptide, IL-6, and TNF-α had a significantly higher clear-cell RCC risk compared with those with low levels of these biomarkers [44]. The implications of this evidence further support the role of chronic inflammation and the insulin pathway in the pathogenic growth of RCC.

Furthermore, some evidence indicated that fatty acid synthase (FASN) expression was correlated with worse outcomes in RCC [45,46]. In this regard, data from The Cancer Genome Atlas dataset showed that *FASN* gene expression was inversely correlated with BMI (*p* = 0.034), and median OS was longer in patients with low *FASN* expression (36.8 v 15.0 months; *p* = 0.002). Of note, FASN immunohistochemistry positivity was significantly higher (*p* = 0.015) in poor-risk patients (48%) compared with intermediate (34%) and good-risk (17%) patients [47].

The association between metabolic factors and the risk of RCC has also been investigated. In a large prospective study within the Metabolic Syndrome and Cancer Project (Me-Can), clinical data on blood pressure, BMI, blood glucose, cholesterol, and triglycerides were collected from a population of 560,388 subjects. Taken separately, high glucose and triglycerides levels, as well as high BMI and blood pressure were correlated with an increased risk of RCC among men, while only BMI had a significant correlation among women. Interestingly, blood pressure and triglycerides among men and BMI among women were independently associated with an increased risk of RCC; however, no biological interaction was discovered between the factors assessed and RCC risk [48]. These data underline the importance of certain metabolic factors, although is indisputable that further studies are needed to shed light on the interplay between metabolic profiles and the risk of RCC development.

## 6. Obesity and Response of RCC to Medical Therapies

Currently, while the therapeutic options for metastatic RCC patients have been expanded with the introduction of immunotherapies and new TKIs, on the other hand, the lack of validated predictive factors to accurately guide oncologists’ decisions is still an unsolved challenging issue.

Several studies have examined the prognostic/predictive role of obesity in metastatic RCC patients undergoing systemic therapy (Table 1). In a heterogeneous group of 116 metastatic RCC patients treated with antiangiogenic agents (mainly sunitinib) as first-line treatment, Steffens et al. retrospectively observed that visceral fat area (VFA) and subcutaneous fat area (SFA) were predictive biomarkers of longer PFS and OS, while BMI and body surface area (BSA) were not [49]. In contrast, in metastatic RCC patients receiving targeted agents (*n* = 64) or immunotherapy (*n* = 49), VFA and SFA were significantly correlated with shorter PFS and OS [50]. More recently, Mizuno and colleagues [51] used BMI and VFA as obesity indices to evaluate the correlation with survival outcomes in 114 patients with metastatic RCC and receiving systemic therapy; most study patients were treated with VEGFR-TKIs, mainly sunitinib and sorafenib, and a few patients received temsirolimus and cytokine. The authors demonstrated that VFA was significantly associated with improved PFS (*p* = 0.007) and OS (*p* = 0.0001); in addition, VFA resulted to be an independent predictive factor of survival [51]. The implications of BMI on OS and on treatment outcomes were investigated in two large metastatic RCC patient populations receiving anti-VEGF receptor and mTOR inhibitors. Both in the International Metastatic Renal Cell Carcinoma Database Consortium (IMDC) cohort including 1975 patients and in the external validation cohort of 4657 patients, high BMI was correlated with a more favorable OS compared to patients with low BMI. This result was maintained when matching with IMDC prognostic criteria. The authors concluded that a high BMI may be a prognostic factor of better survival outcomes in metastatic RCC patients who received targeted therapy, both in first- and second-line settings [47]. This is similar to the results published by Choueiri and his colleagues [52], who reported a survival advantage for obese patients with metastatic RCC treated with targeted therapies. In this study, 475 patients who received sunitinib (61%), sorafenib (30%), or bevacizumab (9%) were stratified according to BMI. Median OS was 1 year longer in obese RCC patients (*p* = 0.0001), and obesity was an independent factor for better OS in the multivariate analysis adjusting for the Heng prognostic risk groups (HR = 0.67, *p* = 0.01) [52].

Of interest, in 71 metastatic RCC patients under first-line sunitinib treatment, Ishihara and co-workers [53] retrospectively evaluated the impact of cancer cachexia on survival through two indicators, namely sarcopenia and the modified Glasgow prognostic score (mGPS). Selected parameters for muscle tissue computed tomography-scan derived and matched with BMI allowed to define the condition of sarcopenia; the mGPS was instead assessed through the level of C-reactive protein (CRP) and albumin (high mGPS was equal to CRP > 1.0 mg/dL and albumin < 3.5 g/dL). The results showed that sarcopenia was markedly correlated with shorter median PFS and median OS, in contrast to non-sarcopenic patients, and that patients with higher mGPS significantly displayed inferior PFS and OS. In the multivariate analysis, sarcopenia and mGPS were independent predictors of reduced PFS and OS, respectively [53].

In February 2019, at the American Society of Clinical Oncology Genitourinary Symposium, Martini et al. [54] presented their retrospective results of 65 RCC patients treated with cabozantinib. The majority of patients had a BMI > 25, and almost all were classed as intermediate/poor-risk. The results showed that higher BMI was significantly correlated with longer OS, without showing significant differences in terms of gastrointestinal and other drug-related toxicities [54].

The relationship between obesity and the outcome of patients treated with immune checkpoint inhibitors has also been investigated. In this regard, Lalani et al. [55] retrospectively collected data from 147 patients treated with immunotherapy alone or in combination with targeted therapies. At a median follow-up of 25 months, patients with high BMI (>25) had longer median OS compared with low BMI (<25) patients (34 vs. 16 months, 2-year OS 61 vs. 42%, *p* = 0.016), while only a non-significant trend was registered for PFS (8.2 vs. 5.9 months, *p* = 0.400). Interestingly, patients who presented a BMI reduction during immunotherapy from >25 to <25 had shorter OS (HR = 2.25, 0.94–5.35) than did those with no BMI changes.

In this regard, a cooperative Italian group headed by De Giorgi carried out a post hoc analysis of a large population of metastatic RCC patients treated with nivolumab immunotherapy within an Italian Expanded Access Program to investigate the prognostic relevance of BMI along with measures of inflammation (neutrophil-to-lymphocyte ratio, systemic immune index (SII), platelet-to-lymphocyte ratio). In univariate and multivariate analyses, BMI was found to be significantly associated with OS; in particular, patients with normal BMI (<25 kg/m^2^) along with SII > 1.375 had shorter OS than did patients with no risk factors. The authors concluded that BMI was an independent prognostic factor in nivolumab RCC patients [56].

The relevance of BMI as a risk factor does not routinely place it as a predictor of prognosis in clinical settings. Only recently, in a phase III trial comparing avelumab plus axitinib versus sunitinib in previously untreated metastatic RCC patients, BMI appeared in a subgroup analysis. However, this was not a preplanned statistical analysis but a post hoc exploratory analysis. The combination with avelumab plus axitinib performed better than sunitinib regardless of BMI status (<25 or >25) [57].

## 7. Concluding Remarks

The selected clinical data from metastatic RCC patients with obesity or increased BMI that we present in this work seem to generally indicate a promising survival benefit, both under targeted therapies and using immunotherapies.

Currently, treatment decision-making in metastatic RCC patients does not take into account whether a patient is habitually obese or non-obese. Indeed, obesity as well as other clinical features including age, gender, and ethnicity have not been prospectively validated as a predictive biomarker of response to therapy. Together with the lack of molecular predictive factors and, as is well known, the controversial role of PD-L1 expression in patients treated with immune checkpoint inhibitors, there emerges a dramatic need for prospective trials aimed at definitively assessing the influence of these factors on the outcomes of RCC patients. Whether BMI or VFA/SFA are the best factors for treatment monitoring in RCC patients is still an open and intriguing question.

Some researchers have defined the relationship between obesity and RCC as an “apparent obesity paradox” [47]. In such sense, it has been proposed that the correlation between high BMI and longer OS can be explained considering the heterogeneity of RCC and, in particular, less aggressive histological subtypes [58]. This view has been further reinforced by the demonstration in obese patients of downregulated FASN expression [47].

In recent years, microbiota as emerged has a promising therapeutic target in patients with lung and RCC who receive immunotherapeutic approaches. This is mainly based on the results published by Routy et al. [59], reporting that primary resistance to immune checkpoint inhibitors may be associated with abnormal gut microbiome composition, as demonstrated by the enhanced response to immunotherapy obtained via the transplantation of fecal microbiota from responders into germ-free or antibiotic-treated mice. In this setting, the close relationship between the composition and function of gut microbiota and obesity seems to merit careful consideration. Notwithstanding, the link between microbiota and obesity or obesity-related conditions (including type 2 diabetes) has not yet been completely clarified. Among the proposed mechanisms, altered integrity of the gut barrier and of the capacity to extract energy from foods, together with changes in the modulation of chronic inflammation, seem to be the most probable candidates [60].

Promising results in in vivo models have recently been observed with novel therapeutics targeting adipocytes, ASCs, and adipose endothelium [61,62], paving the way for tailored therapies for obese patients. In the near future, the challenge will be that of targeting cellular pathways of WAT growth prone to cancer induction and progression in an attempt to reduce cancer risk [63]. Novel evidence has recently reinforced the role of insulin as risk factor; however, it appears clear that there is an unequivocal need to understand how the insulin pathway increases the risk of RCC and to discover further biological pathways as well as obesity-related mechanisms involved in RCC growth. Of interest, cellular pathways involved in the switch of cancer cells to lipid metabolism could be potential targets along with conventional therapies. Areas of investigation for explaining how obesity affects clinical outcomes should consider both pharmocokinetics and drug bioavailability, in addition to the concomitance of cardiovascular disease and diabetes. It is conventionally known that obesity elicits a biological condition of inflammation with consequent increases in TNF, interleukins, C-reactive protein, etc. However, it is important to note that not all obese patients express inflammation of the adipose tissue or metabolic complications.

To conclude, since the incidence of obesity is rising, and its implications can have serious medical consequences, the interplay between obesity and cancer warrants further investigation by the scientific community worldwide. Although several studies are focused on the treatment and prevention of RCC, the first stones on the path toward targeted therapy development for RCC patients are only just beginning to be laid.

## Figures and Tables

**Table 1:** Obesity biomarkers and RCC response to medical therapies.

First Author (Year)	Trial Design	SS	Drugs Class	Obesity Biomarkers	Outcome
Albiges (2016) [47]	R	6632	TKIs	BMI	BMI ↑ = improved OS
Steffens (2011) [49]	R	116	TKIs	VFA and SFA	improved PFS/OS
Ladoire (2011) [50]	R	113	TKIs or ICI	VFA and SFA	shorter PFS/OS
Mizuno (2017) [51]	R	114	TKIs	BMI and VFA	VFA: improved PFS/OS
Choueiri (2019) [52]	R	475	TKIs	BMI	Obese patients = improved OS
Ishihara (2016) [53]	R	71	TKIs	Sarcopenia and mGPS	shorter PFS/OS
Martini (2019) [54]	R	65	TKIs	BMI	BMI ↑ = improved OS
Lalani (2019) [55]	R	147	TKIs + ICI	BMI	BMI ↑ = improved OS
De Giorgi (2019) [56]	R	313	ICI	BMI	normal BMI = shorter OS
Motzer (2019) [57]	R	886	ICI + TKIs	BMI	no interference on survival

Abbreviations: SS, sample size; R, retrospective; TKIs, tyrosine kinase inhibitors; VFA, visceral fat area; SFA, s.c. fat area; ICI, immune checkpoint inhibitors; BMI, body mass index; mGPS, modified Glasgow prognostic score; PFS, progression-free survival; OS, overall survival; ↑, high.
